# Cationic Lignin Polymers as Flocculant for Municipal Wastewater

**DOI:** 10.3390/polym13223871

**Published:** 2021-11-09

**Authors:** Courtney Moore, Weijue Gao, Pedram Fatehi

**Affiliations:** Green Processes Research Centre and Biorefining Research Institute, Lakehead University, Thunder Bay, ON P7B5E1, Canada; ctmoore@lakeheadu.ca (C.M.); wgao@lakeheadu.ca (W.G.)

**Keywords:** lignin polymerization, flocculation, coagulation, municipal wastewater

## Abstract

The radical polymerization of acid-washed and unwashed softwood kraft lignin with [2-(methacryloyloxy) ethyl] trimethylammonium chloride (METAC) was attempted to investigate the production of lignin-based flocculants for simulated wastewater. The incorporation of METAC onto lignin resulted in a cationic charge density (2.3–3.3 meq/g), increased water solubility (89–96% in neutral pH), and increased molecular weight (70,000–210,000 g/mol) of lignin. The lignin–METAC polymers generated from acid-washed lignin had higher molecular weights than those generated from unwashed lignin. The lignin–METAC polymers showed lower resistance to thermal decomposition than unmodified lignin due to the inclusion of PolyMETAC. The unmodified acid-washed lignin samples did not significantly affect the COD of the wastewater, while the unmodified unwashed lignin samples contributed to the COD, implying that unmodified lignin was not suitable for wastewater treatment. The flocculation of wastewater with lignin–METAC led to the chemical oxygen demand (COD) reduction of 17–23% and total organic carbon (TOC) drop of 51–60%. The lignin–METAC polymer with the highest molecular weight (produced from acid-washed lignin) reached the highest COD removal, while lignin–METAC polymer with the highest charge density (produced from unwashed lignin) reached the highest TOC removal. Focused beam reflectance measurement (FBRM) studies revealed that the lignin–METAC polymer produced from acid-washed lignin with a high molecular weight generated larger and more flocs in wastewater than the lignin–METAC polymer produced from unwashed lignin. The comparison of theoretical and experimental dosages required for neutralizing the charges of wastewater demonstrated that charge neutralization was the main flocculation mechanism, although a bridging mechanism was also involved for component removals from wastewater. The use of 1 mg/L of alum along with 65 mg/L lignin–METAC in a dual coagulation–flocculation system led to higher average phosphorous (42%) and COD (44%) removals than the singular flocculation system only using 65 mg/L of lignin–METAC (with phosphorous removals of 3.4% and COD removals of 18.7%). However, lignin–METAC flocculant slightly increased the ammonia–nitrogen content in both singular flocculation and dual coagulation–flocculation systems due to the residual ammonia content of lignin–METAC. The coagulation–flocculation system determined that the use of lignin–METAC (65 mg/L) could reduce the alum dosage significantly while maintaining a similar organic content reduction of 44% for wastewater.

## 1. Introduction

Coagulants and flocculants are often considered the second most expensive operational costs of municipal wastewater treatment facilities next to electricity [1,2]. Organic removal from municipal wastewater with polymer flocculants often reaches >90% efficiency [3,4]. However, polymeric flocculants are relatively inefficient in phosphorous removal [5]. A high organic removal and satisfied phosphorous removal (>95%) via coagulation with alum, aluminum phosphate, and iron salts have been documented in the literature [6]. However, coagulation often requires high dosages, resulting in the production of large volumes of sludge and high disposal and landfilling costs [4]. In this regard, coagulation–flocculation, in which polymer flocculants and coagulants are used together, is often utilized for municipal wastewater treatment to reduce the coagulant dosage while still achieving effective organic removal [4]. As common flocculants and coagulants are often considered toxic, corrosive, and non-biodegradable [4], a more cost-effective and environmentally friendly flocculant would benefit wastewater treatment facilities greatly, as it could reduce the secondary contamination of wastewater.

Globally, the most underused renewable resource is lignin [7]. Lignin composes up to 30% of plant biomass and is a waste product of the pulp and paper industry [7]. An increase in environmental concerns and awareness has generated significant interest in the utilization of this polymeric macromolecule. An increased interest in the application of lignin as a flocculant has emerged due to its high molecular weight, functionality, and availability [8,9,10]. In addition, the reports on the complexation of lignin and metals, such as copper, cobalt, chromium, lead, and zinc, have further increased interest in its application in wastewater treatment [11,12,13,14]. The flocculation efficiency of lignin in wastewater produced by the mining, textile, and pulp and paper industries, as well as model azo-dye wastewaters, were assessed in the past [8,9,15]. However, industrial wastewaters usually contain specific chemical streams at high strength from a specific industry or process. Considering the large difference in the properties of these wastewater constituents, the flocculants used for specific industrial wastewater are not necessarily efficient for other wastewaters. Municipal wastewater is the most abundant type of wastewater with low-strength waste streams, which is characterized by low organic strength and high particulate organic matter content [16]. To our best knowledge, no reports are available for evaluating the efficiency of lignin-based flocculants in municipal wastewater.

Cationic lignin has been prepared in the literature following various methods including the Mannich reaction with amine monomers [9,17], graft copolymers with various cationic monomers [8], and etherification with amines [18]. However, these cationic lignin derivatives were produced from multiple reaction steps, which enhanced the complexity of their production. More importantly, short-chain amines (e.g., dimethylamine, hexane-diamine) used in these modifications led to their relatively low molecular weights, which would weaken their flocculation performance. Graft polymerization with cationic monomers could increase the molecular weight of lignin, which could lead to an increased flocculation performance of lignin. Previous studies conducted by Wang et al. outlined the graft-polymerization of lignin with [2-(methacryloyloxy) ethyl] trimethylammonium chloride (METAC) and the exceptional flocculation performance of the generated lignin–METAC in anionic azo-dyes and kaolin particles [19,20]. Additionally, this reaction does not require an expensive organic solvent system or additives and occurs in a mildly acidic (pH 4) aqueous solution, lowering the production costs as it forgoes complex solvent recovery processes [21]. Finally, the radical initiator, i.e., potassium persulfate, is readily available, inexpensive, and efficient at low dosages, contributing to the attractiveness of this modification method [22,23]. Therefore, the lignin–METAC polymer could be readily produced for wastewater treatment systems.

However, the variability of lignin in its complex structure and properties is largely influenced by its origin (e.g., wood species and their growing conditions) and the processes/conditions of lignin extraction/production from biomass and pulping processes. Lignin impurities such as sugars, silicates, sulfur, ash, and proteins, originating from the raw biomass and industrial pulping processes, pose significant limitations to lignin modification reactions [24,25]. Researchers have suggested that high levels of purification are required when lignin is used in the synthesis of low molecular weight substances or polymers [24,25]. These substances have been recorded to form undesirable by-products and lower yields during the modification process [24,25]. Lignin purification is often accomplished by acid washing via the addition of sulfuric acid resulting in lignin precipitation, which is then separated from solution via filtration or ultra-centrifugation [24,26,27]. In this study, different industrial lignin samples (i.e., acid-washed and unwashed) were received and examined for generating cationic lignin polymers. These samples were subsequently applied as a flocculant for synthetic municipal wastewater treatment. The key objectives of this study were to (1) determine if commercially induced acid-washed and unwashed lignin would have different polymerization performance; (2) synthesize a lignin–METAC polymer to be used as a flocculant in municipal wastewater; and (3) assess the efficiency of lignin–METAC polymer in model wastewater.

## 2. Materials and Methods

### 2.1. Materials

Four commercially produced softwood kraft lignin samples were received from FPInnovations; two of the samples were washed with H_2_SO_4_ to pH 2 (AWL1 and AWL2) and two were not washed (UWL1, UWL2). Chloroform-d 99.8% was purchased from Acros Organics and used for the NMR analysis. Instant skim milk powder (with vitamins palmitate and vitamin D_3_ was received from Pacific^®/MD^, and ethanol 95 vol % was purchased from Fisher Scientific, Ottawa, Canada). [2-(Methacryloyloxy) ethyl] trimethylammonium chloride (METAC), 2-chloro-4,4,5,5-tetramethyl-1,3,2-dioxapholane 95%, chromium (III) acetylacetonate, polydiallyldimethyl ammonium chloride (PDADMAC with a M_w_ of 100–200 kg/mol), potassium persulfate (>99.0%), cyclohexanol, pyridine anhydrous 99.8%, sodium hydroxide (reagent grade), soluble starch, ASC reagent grade, Genapol^®^ X-100, sunflower *helianthus annuus* seed oil, zinc chloride (anhydrous, >97%), nickel (II) sulfate hexahydrate (99%), urea, potassium phosphate monobasic powder (>99.0%), peptone from casein and other animal proteins, sodium dodeylbenzene sulfonate technical grade, ammonium chloride, meat extract, chromium (III) nitrate nonahydrate (99%), copper (II) chloride dihydrate, magnesium phosphate dibasic trihydrate, and diatomaceous earth powder, alum, sodium hypochlorite, sodium salicylate, sodium nitroferricyanide, sodium nitrate, ammonium persulfate, ammonium molybdate, potassium antimonyl tartrate, ascorbic acid, and sulfuric acid (95–98 wt %), reagent grades, were all purchased from Sigma Aldrich, Oakville. Canada. Anionic polyvinyl sulfate (PVSK with the M_w_ of 100–200 kg/mol; 97.7%) was purchased from Wako Pure Chem. Ltd., Osaka, Japan.

### 2.2. Synthetic Wastewater

The synthetic municipal wastewater was simulated based on the wastewater model used by Nopens et al. [28]. Briefly, the synthetic municipal wastewater was prepared in tap water by dissolving the following components: peptone (1.5 mg/L), sodium acetate (120 mg/L), meat extract (1.5 mg), glycerol (40 mg/L), starch (50 mg/L), sunflower seed oil (30 mg/L), milk powder (120 mg/L), urea (75 mg/L), NH_4_Cl (11 mg/L), MgHPO_4_ (25 mg/L), KHK_2_O_4_P (20 mg/L), sodium dodecylbenzene sulfonate (10 mg/L), Genapol^®^ X-100 (10 mg/L), diatomaceous powder (10 mg/L), starch (80 mg/L), calcium chloride (5 mg/L), FeSO_4_ (10 mg/L), and trace metals (i.e., 0.24 mg/L CuCl_2_, 0.34 mg/L Cr(NO₃)₃, 0.15 mg/L NiSO₄, 0.09 mg/L ZnCl_2_, and 0.01 mg/L EDTA).

### 2.3. Lignin METAC Polymer Synthesis

Lignin samples (5.00 g, 27.74 mmol), METAC (11.53 g, 44.340 mmol), and deionized water (100 mL) were added to 500 mL three-neck round-bottom flasks and mixed (300 rpm) overnight. The pH was adjusted (≈4), and the sample was degassed with nitrogen via a Schlenk line. Potassium persulfate solution (2 mL, 138.7 mmol/L) was used as an initiator, and the reaction progressed for 3 h at 80 °C under an inert atmosphere. Upon completion, the sample was cooled, neutralized (pH ≈7), and purified via ethanol precipitation. The precipitate was dried at 105 °C overnight and stored for further testing.

### 2.4. Charge Density, Solubility, and Elemental Analyses

The solubility of the samples was determined following a previously established method [29,30]. The charge density of the samples was determined using a Particle Charge Detector (Mutek PCD 04, BTG Instruments GmbH, Herrsching, Germany) as described in the past [29,30].

An elemental analyzer, Elementar Vario El Cube, was used for determining the carbon, hydrogen, nitrogen, and sulfur contents of the samples (2 mg) using a previously described method [31]. The nitrogen content of the samples was used to calculate the grafting ratio (GR) as described in the past [19]. The metal analysis of the samples was carried out by inductively coupled plasma atomic emission spectroscopy (ICP-AES) using a Varian Vista Pro Radial analyzer, equipped with a cyclonic spray chamber and a Meinert TR-32-K2 nebulizer.

### 2.5. Hydroxyl Group Analysis

Unmodified lignin (36.6 mg) was added to a 10 mL vial and dissolved in 0.5 mL of a 5:8 *v/v* deuterated chloroform/pyridine solution. Cyclohexanol (35 μL, 20 mg/mL) and chromium (III) acetylacetonate (50 μL, 5 mg/mL) were added to the lignin solution as the internal standard (IS) and relaxation agent (RA), respectively. Finally, 100 μL of phosphorylating reagent (PR), 2-chloro-4,4,5,5-tetramethyl-1,3,2-dioxapholane, was added to the lignin solution and allowed to mix (50 rpm) for 45 min before transferring to a 5 mm NMR tube. The NMR spectra were acquired using a Unity Inova 500 MHz nuclear magnetic resonance (NMR) (Varian Inc., Palo Alto, CA, USA)). Due to the high water solubility of the unwashed samples of UWL1 and UWL2, it was not possible to analyze them via ^31^P NMR. The spectra were acquired with 512 scans, and a 5 s delay between 90° pulses. This method is in agreement with methods previously established in the literature [32,33].

### 2.6. Molecular Weight Analysis

Lignin derivatives and PMETAC samples (50 mg) were dissolved in sodium nitrate (10 mL, 0.1 mol/L) and filtered with a 0.2 µm pore-size nylon filter. Gel Permeation Chromatography (GPC) analysis was conducted for determining the molecular weights of the samples using a Malvern GPCmaz VE2001 Module + Viscoteck TDA305 multi-detector (UV, IR, low angle and right-angle laser) (Malvern Panalytical Ltd., Malvern, UK), which was equipped with PolyAnalytic PAC103 and PAC101 columns and 5% acetic acid solution solvent/eluent. A flow rate of 0.50 mL/min and a column temperature of 35 °C was used, and pullulan (47,300 g/mol) was used as standard [19].

### 2.7. Thermal Gravimetric Analysis (TGA)

An Instrument Specialist i1000 series system, Madison, WI, TGA analyzer was used for measuring the thermal behavior of the samples under nitrogen gas following a previously established method [29]. The weight-loss rates of the lignin samples (5–7.4 mg) were determined in the range of 25–650 °C with a nitrogen gas flow of 30–40 mL/min and a heating rate of 10 °C/min. The reactivity index (R) was calculated according to Equation (1):

(1) $$R=\left(\frac{1}{w_{0}}\right){\left(\frac{\mathrm{dw}}{\mathrm{dt}}\right)}_{\max}$$

where R denotes the reactivity index (%/mg·s), w_0_ denotes the initial sample mass (mg), and (dw/dt)_max_ denotes the maximum weight loss rate (%/s).

### 2.8. Flocculation and Coagulation of Wastewater

A 40 mL aliquot of the synthetic wastewater was dispensed into a 50 mL centrifuge tube via a syringe. Various dosages (0–150 mg/L) of the unmodified or modified lignin samples were dispensed gravimetrically into the centrifuge tubes, and the sample was inverted 10 times. The sample was mixed in an Innova 3100 (Brunswick Scientific, Edison, NJ, USA) water bath shaker for 15 min and centrifuged in a Thermo Scientific Sorvall ST 16 centrifuge for 10 min at 1500 rpm. The supernatant was decanted into a clean dry tube and saved for further testing as outlined below.

In another set of experiments, alum and modified lignin polymers were added to 40 mL synthetic wastewater together for coagulation–flocculation tests by following the same process stated above. The dosage of alum was 1 mg/L, and the dosage of lignin polymers was 65 mg/L, which was selected as the optimum dosage for treating wastewater with modified lignin.

### 2.9. Chemical Oxygen Demand (COD) and Total Organic Carbon (TOC) Analysis

The COD analysis of the wastewater samples was carried out using a COD test kit (CHEMetrics, Midland, VA, USA) and a COD CR2200 WTW reactor following the experimental conditions stated in another report [34]. The TOC of the wastewater samples was conducted by a Vario TOC Cube (Elementar Analysensystem Gmb, Langenselbold, Germany) [34].

### 2.10. Total Ammonia and Phosphorous Analysis

Total ammonia as nitrogen (denoted as Ammonia-N) of the wastewater before and after the treatment was determined colorimetrically with a Skalar autoanalyzer following the modified ammonia–silicylate Berthelot reaction method (APHA standard methods, 1999). A sodium hypochlorite stock solution was prepared by dissolving hypochlorite (1 mL) and sodium hydroxide (0.5 g) in a 100 mL volumetric flask. A salicylate catalyst stock solution was prepared by dissolving sodium salicylate (10 g), 0.04 g sodium nitroferricyanide (0.004 g), and sodium hydroxide (0.5 g) in a 100 mL volumetric flask. An aliquot of wastewater (1 mL) was pipetted into a test tube, which was followed by 0.25 mL sodium hypochlorite solution (10 mL/L) and 0.25 mL salicylate catalyst solution. The solution was digested for 5–10 min at room temperature and subjected to measurement at the absorbance of 660 nm with a Skalar autoanalyzer.

The total phosphorous contents of the wastewater before and after treatment were identified in accordance with ISO 17025. An aliquot of wastewater (50 mL) and (0.5 g) ammonium persulfate was dispensed into an Erlenmeyer flask. The sample was digested at 97 °C for 30 min and cooled to room temperature. Sodium hydroxide (5 mol/L) was added to neutralize the solution, and the total volume of the sample was adjusted to 50 mL with distilled deionized water. Ammonium molybdate solution (1 mL, 40 g/L), potassium antimonyl tartrate solution (1 mL, 3.42 g/L), and ascorbic acid (1 mL, 0.1 M) were added to the 50 mL solution, producing a blue phosphomolybdic complex that was quantified at 880 nm with a Skalar Autoanalyzer.

### 2.11. Focused Beam Reflectance Measurement (FBRM)

The chord length of the colloids in the solutions was determined using a Particle Track FBRM (Mettler-Toledo, Greifensee, Switzerland) [35]. The wastewater (200 mL) was dispensed into a 500 mL beaker, where a mounted impeller (50 rpm, blade diameter 50 mm) evenly mixed the solution for 10 min. The FBRM contains a particle track E25 probe equipped with a 5 mm scan circle diameter probe and a Class I laser of less than 10 mW with the wavelength of 780 nm in a 25 mm supporting probe, which was positioned 20 mm below the surface of the wastewater. The focal point of the FBRM laser was fixed at −20 μm. An appropriate amount of coagulant/flocculant stock solution was dispensed into the 500 mL beaker. Scans were performed every 3 s at a scan speed of 2 m/s, and the data were collected and analyzed by iC FBRM 4.3 control software into 90 log channels from 1 to 5000 µm. The chord length of the particles was determined by multiplying the rotating velocity of the focused laser beam by the duration time for passing two edges of the particles.

## 3. Results and Discussion

### 3.1. Characteristics of Unmodified Lignin

The properties of the softwood kraft lignin samples are presented in Table 1. The sulfur contained in unmodified lignin originated from the sulfate and reduced sulfides in black liquor of the kraft process that remained with lignin after its extraction from black liquor. The higher sulfur content of washed lignin samples of AWL1 and AWL2 might be associated with residual sulfuric acid that was used for washing the isolated lignin from black liquor [36].

Inorganic elements often separate from the woody material into the black liquor during the kraft pulping process, and eventually, a portion of them remain with the lignin (Table 1 and Table S1). The acid washing of lignin after extraction resulted in a significant decrease in total inorganic content from 10.4–7.61 wt % for unwashed samples to 2.05–1.74 wt % for acid-washed samples, which is consistent with other studies [37,38]. As a result, acid-washed samples had higher organic carbon, hydrogen, and sulfur contents (Table 1). The charge density of acid-washed lignin samples was undetectable due to their low solubility. The salt content can result in the abstraction of hydrogen from aliphatic hydroxyl groups, resulting in anionic lignin, which improved the lignin solubility and charge density of lignin samples for UWL1 and UWL2 [39]. The residual inorganic metals could be bound with the functional groups (e.g., carboxylate, hydroxyl, and sulfonate groups) of lignin.

**Figure 1:**
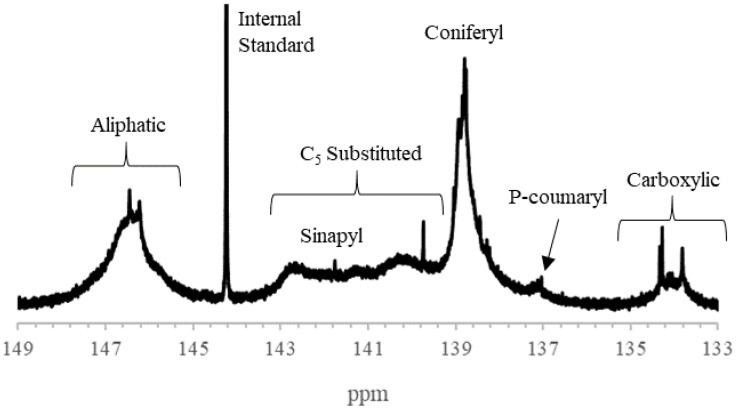
^31^PNMR spectrum of AWL1 with aliphatic (δ 145.4–150.0 ppm), C_5_ substituted (δ 140.0–144.5 ppm), Sinapyl (δ ≈142.7 ppm), conideryl (δ 139.0–140.2 ppm), p-coumaryl (δ ≈137.8 ppm), and carbocyclic (δ 133.6–136.0 ppm).

The quantification of the subunits and hydroxyl group contents of lignin samples was accomplished using ^31^P NMR (Figure 1). Samples AWL1 and AWL2 were determined to have 1.09 and 1.36 mmol/g coniferyl hydroxyl units, respectively (Table 2), which is consistent with other reports about the composition of softwood lignin samples [36]. Softwood lignin samples’ coniferyl alcohol subunits represent the majority of the phenolic hydroxyl groups, whereas hardwood lignin is composed of similar amounts of coniferyl and p-coumaryl units [33].

The reactivity of lignin is largely influenced by its phenolic hydroxyl group content, which facilitates such reactions as the substitution of the hydroxyl hydrogen and electrophilic substitution in the ortho-position [40]. Native softwood lignin is reported to have hydroxyl content that decreases in the order of aliphatic hydroxyl > phenolic hydroxyl > carboxylic hydroxyl [33]. The present data of kraft lignin revealed a different order of phenolic hydroxyl > aliphatic hydroxyl > carboxylic hydroxyl (Table 2). Kraft lignin has been reported to have the highest proportion of phenolic groups than other industrial lignin samples [41]. In the kraft pulping process, sodium sulfide and sodium hydroxide aggressively cleave lignin–carbohydrate bonds, producing a higher phenolic hydroxyl group content [41]. The temperature of the black liquor at the time of acidification can also lead to acidolysis, which can further increase phenolic hydroxyl group formation by cleaving ether linkages [42]. However, the hydroxyl group content of the UWL1 and UWL2 samples could not be determined to compare with the acid-washed samples, as P NMR could be only conducted on insoluble lignin samples.

### 3.2. Characteristics of Lignin–METAC

The lignin samples outlined in Table 1 were polymerized with METAC following the radical graft polymerization method [19]. The reaction scheme for the polymerization of lignin and METAC is shown in Figure S1. The polymerization of lignin and METAC was confirmed by the outcomes of ^1^H NMR (Figure S2), Fourier transform infrared spectroscopy (FTIR), and elemental analyses in our previous publication [19]. The characteristics of the generated products are summarized in Table 3. AM1 and AM2 were generated from acid-washed lignin samples, and UM1 and UM2 were generated from unwashed lignin. The grafting and propagation of METAC monomer on the lignin backbone resulted in a cationic charge density, increased water solubility, and increased molecular weight. The charge density of the lignin sample becomes significantly cationic, 2.3–3.3 meq/g, which was due to the incorporation of the quaternary amine group of METAC monomer [22,43].

All of the modified lignin samples had a higher molecular weight (Mw) than unmodified kraft lignin, which ranges 17,000–26,000 g/mol as reported in our previous studies [19,44]. Consistent with ^31^P NMR analysis of the unmodified lignin, AWL2 had the higher phenolic hydroxyl group content (3.36 mmol/g), which is associated with enhanced polymerization, resulting in a higher charge density than AWL1 [19,41,44]. However, the lower molecular weight of AM2 than AM1 could be attributed to the higher chance of collision or radial coupling termination, as more radicals would be formed at the higher polymerization rate [45].

AM1 and AM2 generated from the acid-washed lignin samples had higher molecular weights than UM1 and UM2 generated from unwashed lignin, demonstrating the lower polymerization efficiency of METAC and unwashed lignin. Generally, as these lignin samples were produced commercially at large scales at different dates, they might have had different physicochemical properties, and thus, it is difficult to determine the exact reason for better polymerization efficiency of acid-washed samples. However, the pH values of the unwashed lignin solutions were tested to be around 9–10. Thus, unwashed lignin samples required pH adjustment before polymerization and had a significantly higher inorganic content before modification (Figure S3) [40,46,47]. However, the high inorganic content of unwashed lignin (Table 1) might have hampered the reactions. Singh and Ormerod investigated the effects of metal ions on free radical reactions with proteins. They concluded that metal salts (specifically FeCl_3_, FeCl_2_, CoCl_2_, HgCl_2_, FeSO_4_, and CuSO_4_) reduced the total radical concentration, resulting in lower product yields and lower grafting efficiencies [46].

### 3.3. Thermal Properties

Thermogravimetric analysis (TGA) was used for determining the weight loss and first derivative weight loss rate of unmodified lignin and lignin–METAC as a function of temperature (Figures S4 and S5). The weight loss below 100 °C is associated with the evaporation of water [25]. The majority of decomposition reactions occur between 200 and 700 °C (Figures S4 and S5). When the unmodified lignin reaches a temperature of 130–200 °C, lignin dehydration occurs, which results in the formation of unsaturated chains, carbon monoxide, carbon dioxide, and methane [25]. This is followed by the fission of methyl-aryl ether bonds, releasing water and formaldehyde in the temperature range of 200–400 °C, and finally the decomposition of phenolic rings via bond saturation, C-C bond cleavage, and methoxy group cracking at 400 °C [25]. Above 500 °C, further rearrangements and condensation of the aromatic structure occur [48], leading to the formation of a significant ash yield (29.0–58.8 wt %) at 650 °C. Unwashed unmodified lignin samples of UWL1 and UWL2 had higher residual mass, which is consistent with their higher inorganic contents, as discussed previously.

The lignin–METAC samples showed lower resistance to thermal decomposition than unmodified lignin, indicating their lower weight loss temperature (Tp), higher decomposition rate (i.e., DTG_max_), and less residual mass (Table 4). In spite of the large differences in the decomposition peak (T_p2_) and ash content for acid-washed and unwashed lignin, their resulting lignin–METAC polymers showed very similar thermal properties. One reason could be that the lignin modification process removes the inorganic species that do not evaporate upon thermal degradation. It can be seen in Figure S3 that the differences in inorganic contents among lignin–METAC polymers were much smaller than that among unmodified lignins. AM2 showed a similar maximum decomposition rate, but a higher ash yield, than AM1, indicating that less METAC was grafted onto AWL2, which is consistent with its lower molecular weight. More pronounced decomposition peaks (T_P2_ and T_P3_) are visible for all modified lignin samples. The first peak (T_P2_) is a result of quaternary ammonia group decomposition, and the second peak (T_P3_) is associated with the decomposition of the METAC backbone [49]. Therefore, the decreased thermal stability of lignin–METAC is associated with the incorporation of PMETAC in the polymer. This observation can be characterized by thermal reactivity index (R) and maximum decomposition rate (i.e., DTG_max_). The thermal reactivity index R is defined as the rate at which the polymer reacts in an oxidizing/reducing atmosphere, describing its decomposition easiness [50]. In addition, the R index and DTG_max_ of lignin–METAC were 50% larger than those of unmodified lignin, indicating that lignin–METAC polymers were much easier to decompose at a higher rate after polymerizing with METAC.

### 3.4. Flocculation of Wastewater by Unmodified Lignin

**Figure 2:**
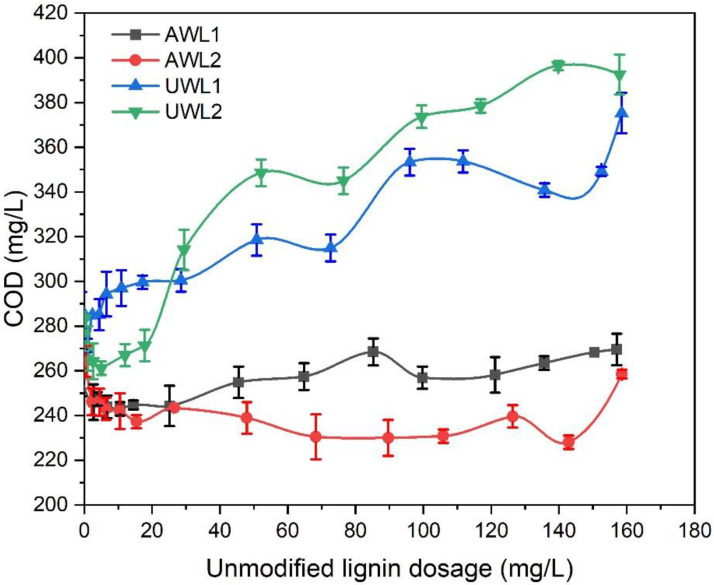
COD of municipal wastewater as a function of unmodified lignin dosage.

The unmodified lignin samples were assessed for their ability to reduce the chemical oxygen demand (COD) of synthetic wastewater samples (Figure 2). The unwashed lignin samples (UWL1 and UWL2) contributed to the COD, which suggested that they remained in the wastewater after centrifugation. Acid-washed lignin samples did not significantly affect the COD of the wastewater. It may be claimed that the low molecular weight, negative charge density, and insolubility of the unmodified acid-washed lignin samples in aqueous systems limited the interaction of lignin with suspended colloids in the wastewater sample.

### 3.5. Flocculation of Wastewater by Lignin–METAC Polymer

**Figure 3:**
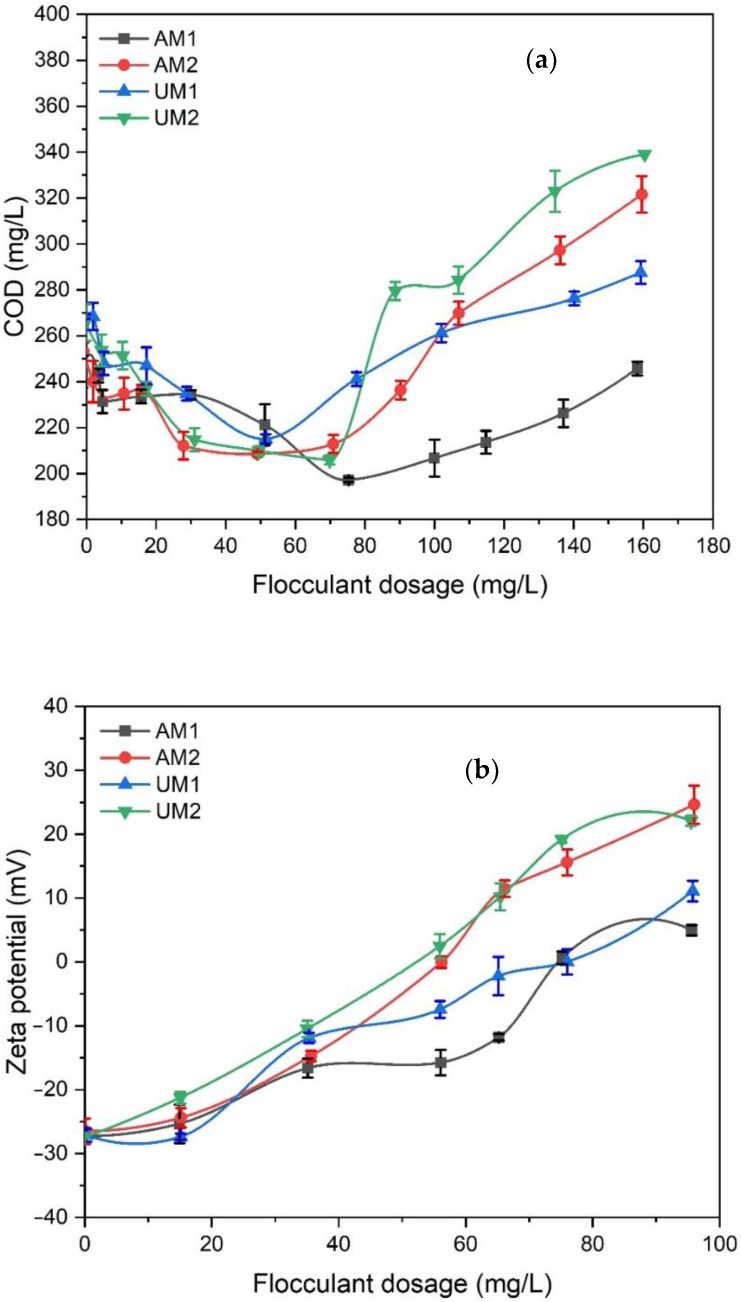
(**a**) COD and (**b**) zeta potential as a function of lignin-METAC dosage for municipal wastewater treatment.

We investigated the effectiveness of the lignin–METAC polymers in treating synthetic wastewater (Figure 3a). The COD of the synthetic wastewater was determined to be 257 mg/L. A decrease in COD was observed as the dosages of lignin–METAC polymers increased. However, high dosages increased the COD of the wastewater, which was probably due to the production of overcharged complexes and thereby restabilizing the colloidal particles. The overdose of the high amount of organic flocculant would also introduce extra COD to the wastewater.

Zeta potential analysis was used for providing an estimate of the optimized flocculant dosage range and insight into the flocculation mechanism (Figure 3b). The reduction in the zeta potential to 0 mV by the flocculants is a result of colloidal charge neutralization through electrostatic attractions between anionic colloids and cationic flocculants [51]. Interestingly, a linear relationship between flocculant dosage and zeta potential is observed (Figure 3b).

The flocculant dosage that results in a zeta potential of zero will be equivalent to the dosage required for the optimum COD removal if the charge neutralization is assumed to be the major flocculation mechanism, which is considered the theoretical flocculant dosage [51]. As seen in Table 5, the theoretical dosage of all lignin–METAC samples was higher than the experimentally optimized dosages (Table 5), which implies that other mechanisms, e.g., polymer bridging, were also involved in the flocculation that improved their efficiency. It is also noticed that despite the higher molecular weight of AM1, the optimum dosage of AM1 was the highest among all the lignin–METAC samples, which could be attributed to its lower charge density (Table 3). Although the lignin–METAC polymers generated from unwashed lignin (i.e., UM1 and UM2) had much lower molecular weight than those generated from acid-washed lignin (i.e., AM1 and AM2), they achieved the same level of COD and TOC removals, which was probably due to their higher charge densities (Table 3). In addition, the TOC removals were much higher than COD removals, indicating that the lignin–METAC flocculants were more efficient in removing organic contaminants than inorganic constituents present in the wastewater because the negative charge and large molecule sizes of organic matters could facilitate their flocculation by charge neutralization and bridging [52,53].

### 3.6. Floc Formation

**Figure 4:**
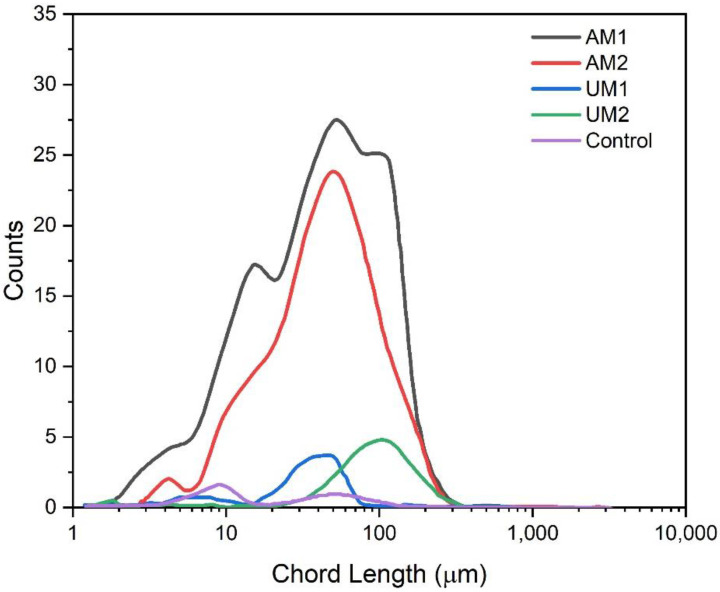
Number of counts as a function of the chord length of flocs formed after the addition of 65 mg/L lignin–METAC polymer in synthetic municipal wastewater.

Focused beam reflectance measurement (FBRM) monitors the flocculation of colloidal systems by characterizing the changes in the average size and size distribution of suspended flocs in wastewater [54]. This method was utilized for characterizing the chord length and number of particles in wastewater after treating with lignin–METAC flocculants (Figure 4). Generally, larger chord length is associated with increased sedimentation rates and improved flocculation performance in the industry [35,55]. FBRM analysis revealed a significant increase in particle (floc) size after the addition of lignin–METAC flocculants. The analysis on lignin–METAC flocculants of AM1 and AM2 revealed extensive floc formation with a significant increase in both floc size (chord length) and number. AM1 and AM2 have higher molecular weight than UM1 and UM2, which would be associated with elevated polymeric bridging, potentially expanding the particle size and counts [56,57].

### 3.7. Dual Coagulation–Flocculation System

Table 6 lists the residual inorganic content, ammonia, phosphorous, and COD in wastewater after treating with the optimized dosages of unmodified lignin, lignin–METAC, or alum for direct coagulation and coagulation–flocculation systems. In practice, a coagulant dosage of alum is usually between 50 and 450 mg/L for municipal and industrial wastewater [58,59]. Direct coagulation with alum (200 mg/L based on the practice of local municipal wastewater treatment plant) resulted in a significant increase in the total inorganic content, which can be potentially harmful due to residual counter ion (sulfate) contamination, where the sulfur content of the wastewater was increased by 492%.

A slight increase in ammonia–nitrogen is observed when the wastewater is treated with a lignin–METAC flocculant, which can be attributed to the residual ammonia content of lignin–METAC. The most reduction in the ammonia–nitrogen content (33.3%) was observed after treating wastewater with alum. Aguilar et al. reported that an 88% albuminoid nitrogen removal could be achieved under optimal coagulation conditions [5]. The reduction in ammonia–nitrogen is often <10%, as the dual coagulation–flocculation processes do not directly remove nitrogen from the solution [5]. The favorable ammonia-nitrogen reduction can only be achieved when wastewater is treated between pH 4 and 7, and the ammonia is in the form of NH_4_^+^ ions, allowing them to join the surface of negatively charged colloids through electrostatic attraction. This pH range is often lower than that of municipal wastewater (pH 6–8) [53].

No COD removal was observed after the treatment with AM1. The flocculation by lignin–METAC only achieved limited phosphorous (3.4%) and COD (18.7%) removals. Wastewater treatment via coagulation–flocculation with alum (1 mg/L) and lignin–METAC (65 mg/L) also significantly decreased the residual phosphorous content (from 8.5 to an average of 5.1 mg/L) when compared with singular flocculation by AM1 only. The maximum phosphorous removal (77%) was achieved via singular coagulation with alum (200 mg/L). The higher rates of phosphorous removal (≈100%) have been achieved in the literature with an increased alum dosage (7600 mg/L) and decreased pH (≈5) [5]. These conditions allow for the chemical precipitation of orthophosphates, which is a mechanism not often favored in municipal wastewater treatment due to required pH adjustment, and high alum dosage. An effective and similar organic content removal is achieved via singular coagulation with alum (200 mg/L), dual coagulation–flocculation with alum (1 mg/L), and lignin–METAC (65 mg/L).

Studies were conducted to evaluate the efficiency of biobased polymers as a suitable replacement of metal salts for wastewater treatment. The flocculation of municipal wastewater by cationic starch–acrylamide achieved the COD removal of 27% [60]. Cationic tannin-based flocculant (Tanfloc) induced high turbidity (90–100%), BOD_5_ (50–60%), and COD (50–60%) removals, which were comparable to the efficiency of alum and polyaluminum chloride (PAC) [61,62]. It was also reported that the combined coagulation–flocculation process by ferric sulfate (as a coagulant, 250 mg/L) and dicarboxylic acid nanocellulose (as flocculant, 2.5–5 mg/L) resulted in 50% COD removal from municipal wastewater [53], which is comparable with this study.

## 4. Conclusions

The radical graft polymerization of acid-washed and unwashed kraft lignin samples with METAC was attempted, and the products were successfully used as flocculants in synthetic municipal wastewater. The modification of all unmodified lignin converted their anionic charge of −1.7 meq/g to a cationic charge density of 2.3–3.3 meq/g. The modification of the lignin samples decreased their thermal stability. The unmodified lignin demonstrated limited functionality as a flocculant in the wastewater, while the lignin–METAC flocculants achieved the maximum removal efficiency of 60% for TOC. The addition of lignin–METAC polymers produced from acid-washed (AM1 and 2) lignin generated more flocs than those produced from unwashed lignin due to their higher molecular weight. The theoretical and experimental dosage analysis confirmed that charge neutralization was the main flocculation mechanism, even though the bridging mechanism was also involved. Only limited phosphorous (3.4%) and COD (18.7%) removals were achieved by lignin–METAC flocculants on average. In a dual coagulation–flocculation system, the use of 1 mg/L of alum with 65 mg/L AM1 increased phosphorous and COD removals to 42% and 44%, respectively. However, AM1 flocculant slightly increased ammonia–nitrogen content in singular flocculation and dual coagulation–flocculation systems, which can be attributed to the residual ammonia content of lignin–METAC. The potential application of lignin–METAC flocculants could largely reduce the alum dosage for municipal wastewater treatment.

## Figures and Tables

**Table 1:** Properties of unmodified softwood kraft lignin samples.

	Charge Density (meq/g)	Solubility (wt %)	Organic Elements (wt %)						Inorganic Elements (wt %)				
			C	H	N	S	O	Al	Ca	Fe	Na	K	Total Inorganics
AWL1	NA *	7.2	65.2	5.2	0.0	2.4	26.9	<0.01	0.01	<0.01	0.31	0.03	2.05
AWL2	NA	7.5	64.0	5.1	0.0	2.2	27.0	<0.01	<0.01	<0.01	0.18	0.02	1.74
UWL1	−1.77	94	49.5	4.3	0.0	1.4	32.9	0.01	0.08	<0.01	7.51	0.98	10.40
UWL2	−1.78	92	54.1	4.5	0.0	1.7	31.6	0.01	0.02	0.01	5.37	0.63	7.61

* NA: not available.

**Table 2:** Aliphatic, carboxylic, and phenolic hydroxyl group contents of unmodified acid-washed lignin samples (±0.03 mmol/g).

	Non-Phenolic Hydroxyl Groups		Phenolic Hydroxyl Groups				
	Aliphatic	Carboxylic	Coniferyl	Sinapyl	P-Coumaryl	C5 Substituted	Total
AWL1	1.13	0.26	1.09	0.46	0.09	0.96	2.60
AWL2	1.36	0.17	1.36	0.65	0.05	1.30	3.36

**Table 3:** Lignin–METAC polymer molecular weight, solubility, charge density, and grafting ratio.

	Mw (kg/mol)	Solubility (wt %)	Charge Density (meq/g)
AM1	210 ± 90	94	2.3
AM2	140 ± 50	96	3.2
UM1	76 ± 9	90	2.6
UM2	70 ± 20	89	3.3

**Table 4:** Peak weight loss temperature (T_p_), integrated weight loss (DTG_maz_), residual mass (W_R_), and reactivity index (R) of unmodified lignin and lignin–METAC polymers determined by TGA (Figures S4 and S5).

	Peak Temperature (°C)			DTG_max_ (%/°C)	W_R_ (%)	R (%/kg·s)
	T_P1_	T_P2_	T_P3_			
Unmodified Lignin						
AWL1	46.4	437	-	0.173	36.2	27,900
AWL2	41.7	414	-	0.181	30.8	29,200
UWL1	50.3	330	-	0.181	58.8	30,100
UWL2	56.6	331	-	0.184	58.0	29,200
Lignin–METAC polymers						
AM1	58.4	278	437	0.410	11.4	66,200
AM2	67.2	287	441	0.436	23.5	70,300
UM1	58.4	281	449	0.456	18.2	76,000
UM2	58.5	277	444	0.506	15.0	80,200

**Table 5:** Optimum flocculant dosages required to reach a zeta potential of 0 mV.

	Optimum Flocculant Dosage (mg/L)		COD Removal, %	TOC Removal, %
	Theoretical ^1^	Experimental		
AM1	75.4	67	23 ± 5	51 ± 3
AM2	54.1	47	19 ± 5	59 ± 3
UM1	67.0	51	17 ± 5	60 ± 3
UM2	51.3	48	19 ± 5	57 ± 3

^1^ Flocculent dosage required to reach a zeta potential of 0 mV.

**Table 6:** Residual total inorganic content, ammonia, phosphorous, and COD in wastewater after treatment.

	Dosage of Lignin, mg/L	Dosage of Alum, mg/L	Total Inorganic Content, ±0.5 mg/L	NH_3_, ±1 mg·L	Phosphorous, ±0.2 mg/L	COD, ±5 mg/L
Wastewater	0	0	62.1	36	8.8	257
AWL1	65	0	60.2	33	6.0	275
AM1	65	0	61.2	44	8.5	209
Alum + AM1	65	1	59.9	44	5.1	144
Alum	0	200	95.0	24	2.2	145

## Data Availability

Raw data can be available upon request from the corresponding author.

## Supplementary Materials

The following are available online at https://www.mdpi.com/article/10.3390/polym13223871/s1, Figure S1: Polymerization scheme of kraft lignin and METAC, Figure S2: ^1^H NMR spectra of lignin, PMETAC, and lignin–graft–PMETAC, Figure S3: Comparison of total inorganic content of unmodified and modified lignin–METAC, Figure S4: (A) Weight loss as a function of temperature and (B) derivative weight loss as a function of temperature for unmodified lignin samples, Figure S5: (A) Weight loss as a function of temperature, and (B) derivative weight loss as a function of the temperature for lignin–METAC samples, Table S1: Trace elemental analysis of unmodified lignin determined via ICP MS analysis.
