# Supplementary material for: Cationic Lignin Polymers as Flocculant for Municipal Wastewater
Source: Polymers (Basel). 2021 Nov 9;13(22):3871. doi: 10.3390/polym13223871 (PMC8625770; doi:10.3390/polym13223871)
Supplement: Supplementary file 1 [file polymers-13-03871-s001.zip › polymers-1416200-supplementary.pdf]

Supplementary materials

Cationic lignin polymers as flocculant for municipal wastewater

Courtney Moore, Weijue Gao, and Pedram Fatehi *

Green Processes Research Centre and Biorefining Research Institute, Lakehead University, Thunder Bay, ON P7B5E1, Canada; ctmoore@lakeheadu.ca (C.M.); wgao@lakeheadu.ca (W.G.)
* Correspondence: pfatehi@lakeheadu.ca; Tel.: +1-807-343-8697; Fax: +1-807-346-7943

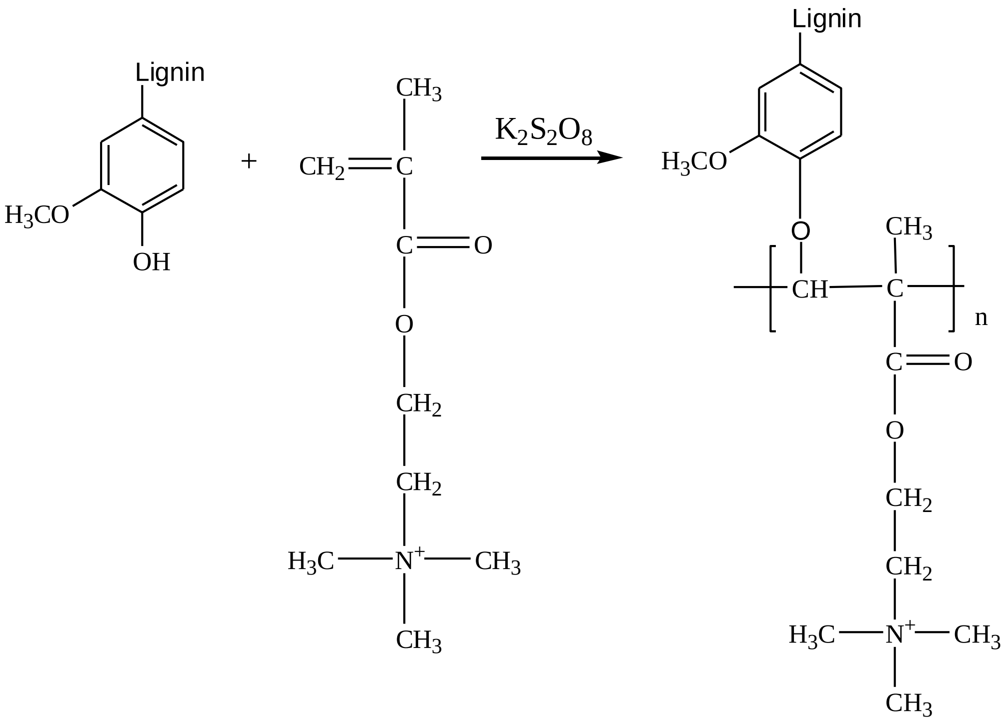

Figure S1. Polymerization reaction of kraft lignin and METAC [19].

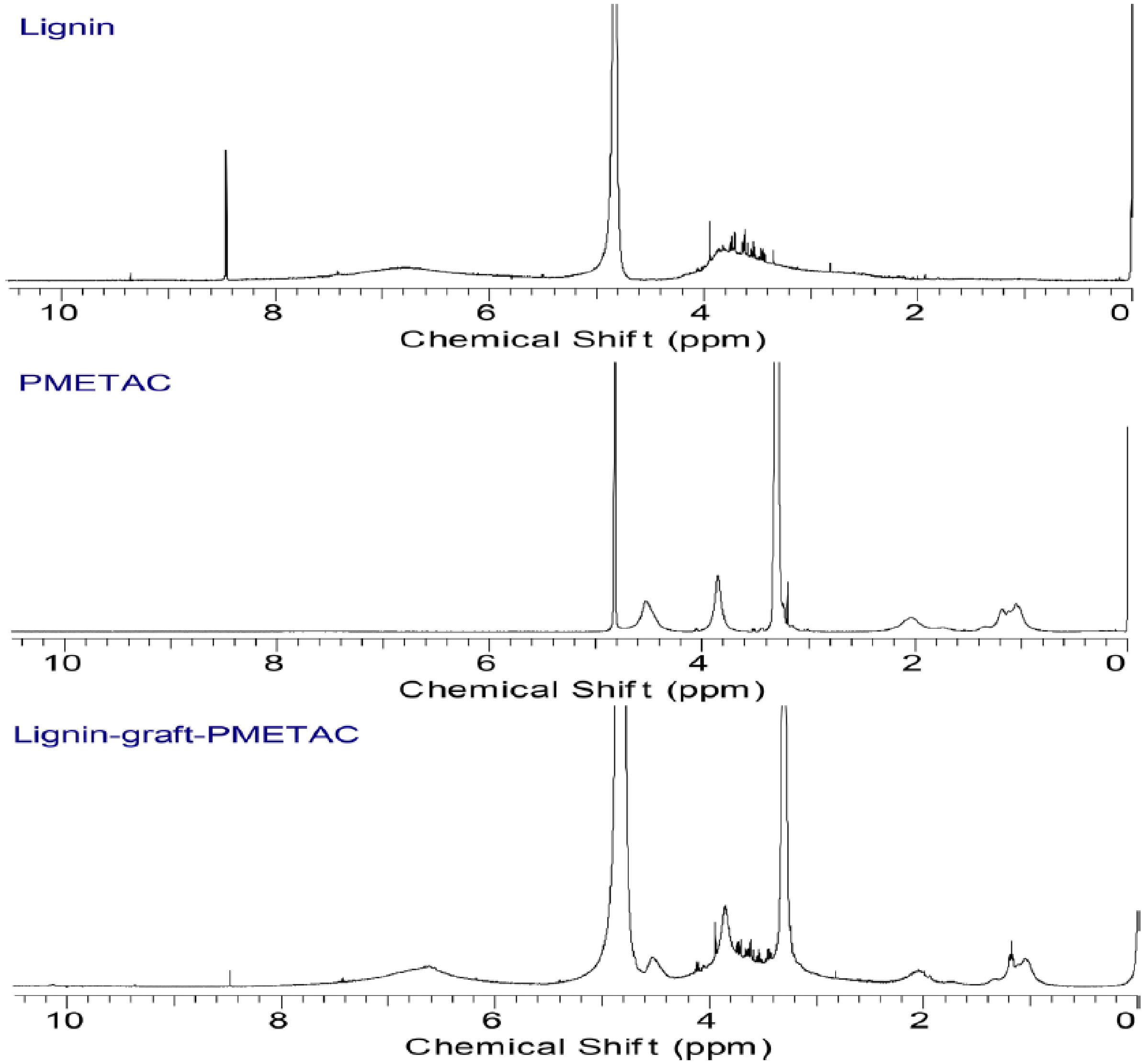

Figure S2. ^{1}H NMR spectra of lignin, PMETAC, and lignin-graft -PMETAC [19].

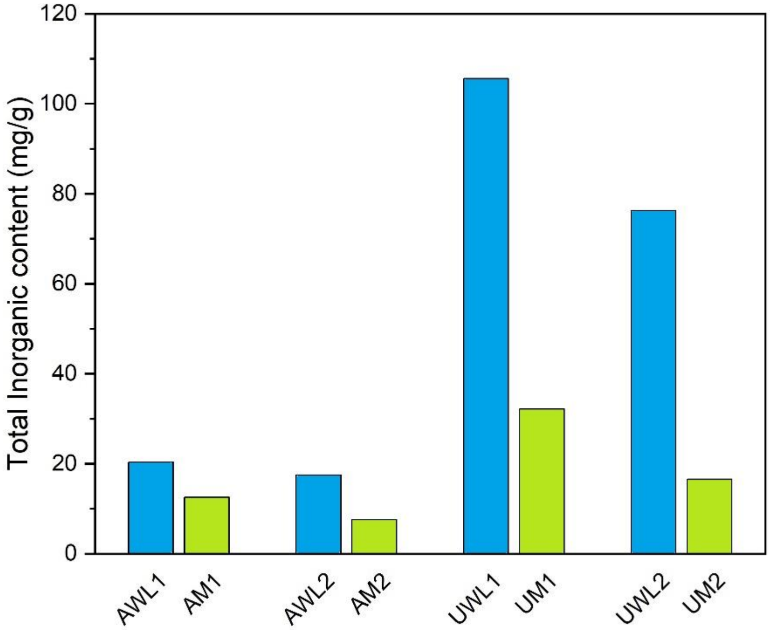

Figure S3. Comparison of total inorganic content of unmodified and modified lignin-METAC.

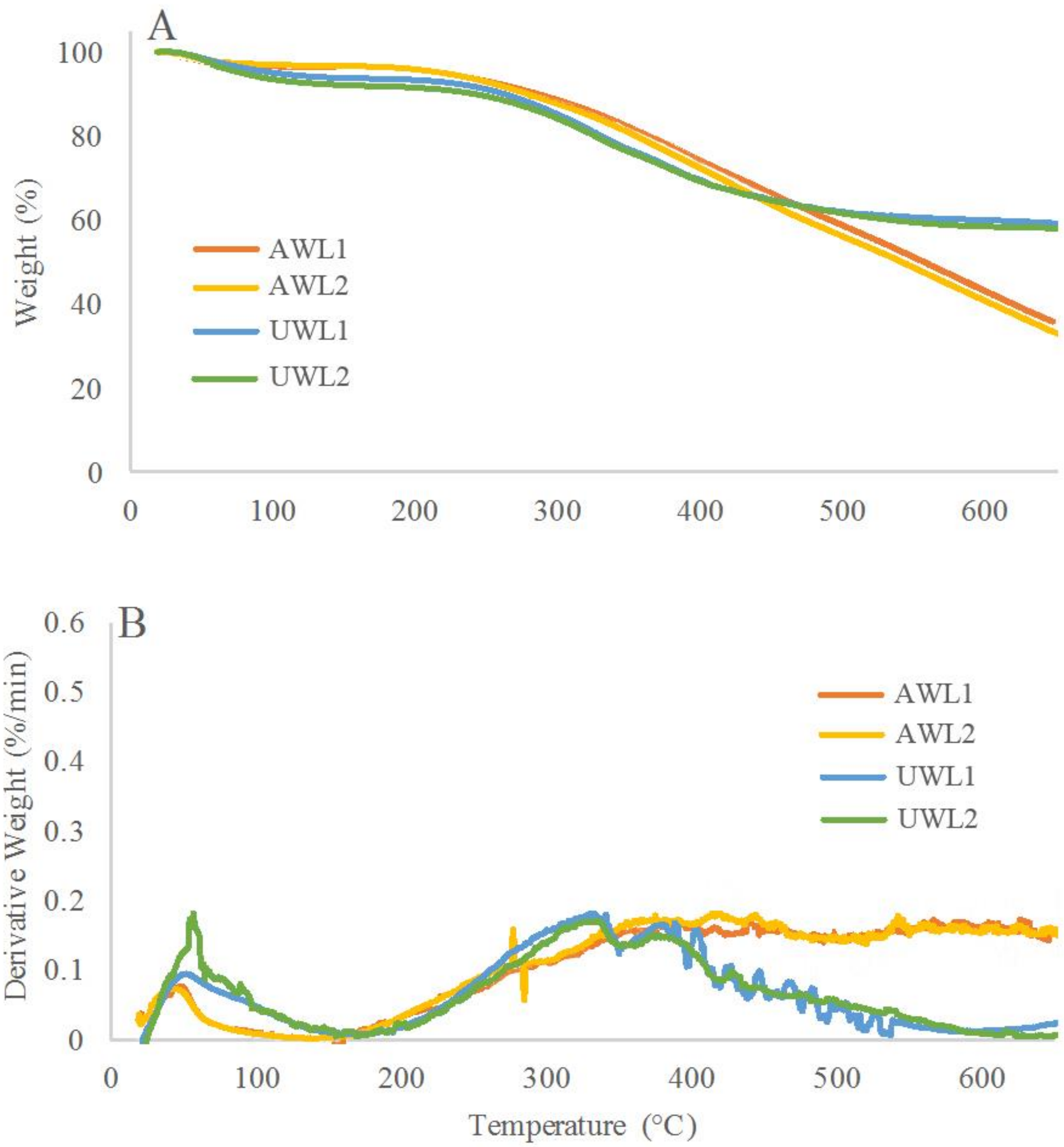

Figure S4. (A) Weight loss as a function of temperature, and (B) derivative weight loss as a function of temperature for unmodified lignin samples.

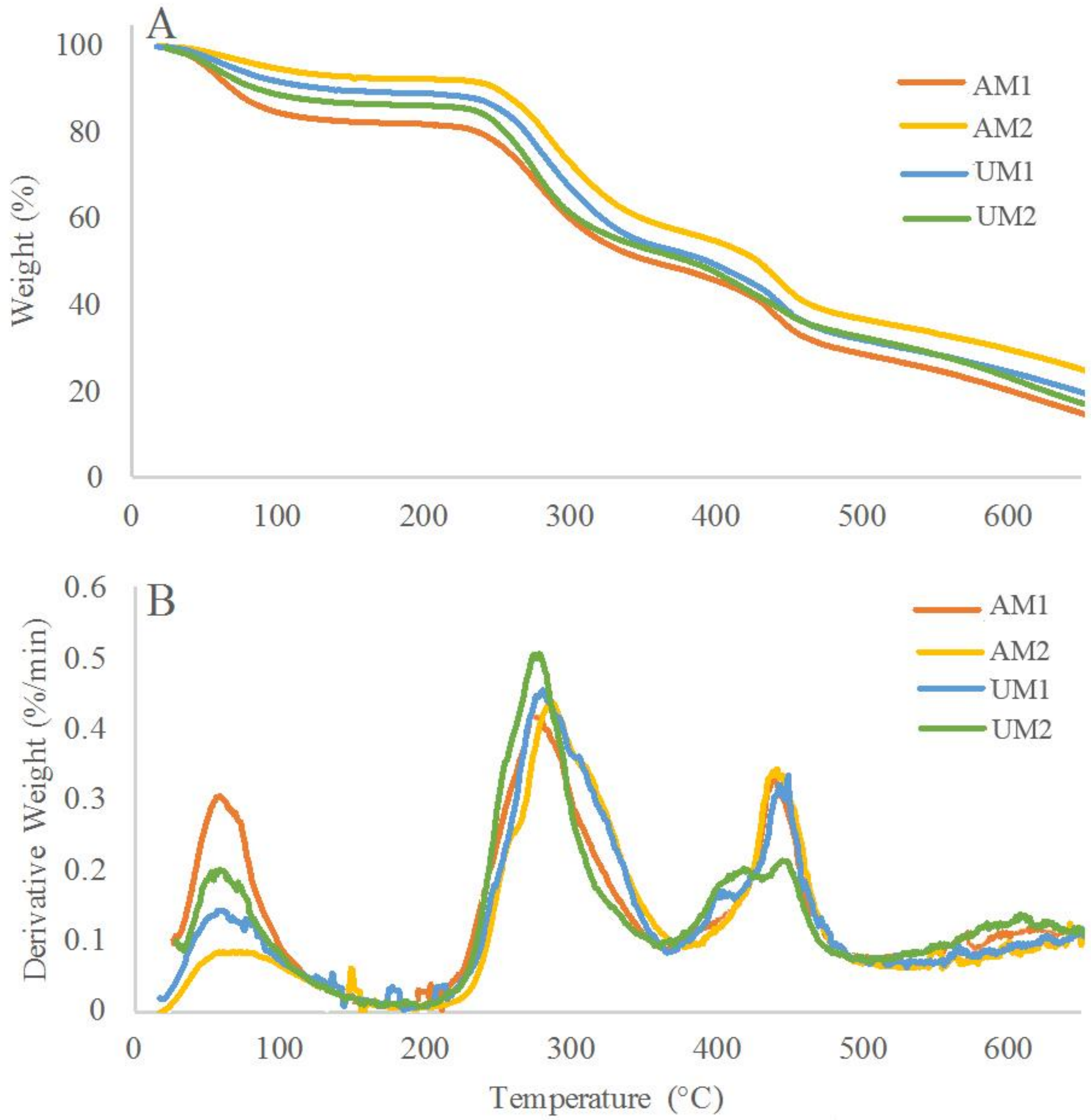

Figure S5. (A) Weight loss as a function of temperature, and (B) derivative weight loss as a function of temperature for lignin-METAC samples.

Table S1. Trace elemental analysis of unmodified lignin determined via ICP MS analysis

	Inorganic Elements (µg/g)													
	**Al**	**Ba**	**Ca**	**Cr**	**Cu**	**Fe**	**K**	**Mg**	**Mn**	**Na**	**S**	**Si**	**V**	**Zn**
**Unmodified**														
**AW L1**	29	0.83	55	1.03	1.23	33.6	289	19.4	5.2	3,052	16,720	270	0.79	2.37
**AW L2**	21	<DL	41	0.86	1.52	9.7	177	10.7	1.8	1,754	15,216	117	2.11	0.53
**UW L1**	68	8.66	843	0.80	0.95	38.1	9772	349.7	185.4	75,144	17,536	447	8.31	17.86
**UW L2**	118	3.64	184	0.26	1.32	58.6	6322	184.1	92.7	53,738	15,221	116	1.71	10.89

<DL: below the Detectable Limits.
